# Quantifying the Contribution of Statins to the Decline in Population Mean Cholesterol by Socioeconomic Group in England 1991 - 2012: A Modelling Study

**DOI:** 10.1371/journal.pone.0123112

**Published:** 2015-04-09

**Authors:** Chris Kypridemos, Piotr Bandosz, Graeme L. Hickey, Maria Guzman-Castillo, Kirk Allen, Iain Buchan, Simon Capewell, Martin O’Flaherty

**Affiliations:** 1 Department of Public Health and Policy, University of Liverpool, Liverpool, United Kingdom; 2 Department of Hypertension and Diabetology, Medical University of Gdansk, Gdansk, Poland; 3 Epidemiology and Population Health Institute of Infection and Global Health, University of Liverpool, Liverpool, United Kingdom; 4 Lancaster Medical School, Lancaster University, Lancaster, United Kingdom; 5 Centre for Health Informatics, Institute of Population Health, University of Manchester, Manchester, United Kingdom; University of Manitoba, CANADA

## Abstract

**Background:**

Serum total cholesterol is one of the major targets for cardiovascular disease prevention. Statins are effective for cholesterol control in individual patients. At the population level, however, their contribution to total cholesterol decline remains unclear. The aim of this study was to quantify the contribution of statins to the observed fall in population mean cholesterol levels in England over the past two decades, and explore any differences between socioeconomic groups.

**Methods and Findings:**

This is a modelling study based on data from the Health Survey for England. We analysed changes in observed mean total cholesterol levels in the adult England population between 1991-92 (baseline) and 2011-12. We then compared the observed changes with a counterfactual ‘no statins’ scenario, where the impact of statins on population total cholesterol was estimated and removed. We estimated uncertainty intervals (UI) using Monte Carlo simulation, where confidence intervals (CI) were impractical. In 2011-12, 13.2% (95% CI: 12.5-14.0%) of the English adult population used statins at least once per week, compared with 1991-92 when the proportion was just 0.5% (95% CI: 0.3-1.0%). Between 1991-92 and 2011-12, mean total cholesterol declined from 5.86 mmol/L (95% CI: 5.82-5.90) to 5.17 mmol/L (95% CI: 5.14-5.20). For 2011-12, mean total cholesterol was lower in more deprived groups. In our ‘no statins’ scenario we predicted a mean total cholesterol of 5.36 mmol/L (95% CI: 5.33-5.40) for 2011-12. Statins were responsible for approximately 33.7% (95% UI: 28.9-38.8%) of the total cholesterol reduction since 1991-92. The statin contribution to cholesterol reduction was greater among the more deprived groups of women, while showing little socio-economic gradient among men.

**Conclusions:**

Our model suggests that statins explained around a third of the substantial falls in total cholesterol observed in England since 1991. Approximately two thirds of the cholesterol decrease can reasonably be attributed non-pharmacological determinants.

## Introduction

Cardiovascular disease (CVD) remains the primary cause of death in the UK and globally [1]. However, UK cardiovascular mortality has been falling consistently since the early 1970s [2]. The two main drivers of this fall have been: reductions in cardiovascular risk factor levels; and improved treatments, both preventive and therapeutic [3].

Serum total cholesterol is one of the main targets for primary and secondary prevention of CVD. In England, the mean total cholesterol of the population has dropped substantially over the past three decades [4]. This fall occurred initially as the result of dietary changes alone [5], but more recently it reflects the interplay between improving diet and increasing statin use [6]. Unlike other cardiovascular risk factors, total cholesterol shows no socioeconomic gradient in young adults and an inverse gradient at older ages, thus more affluent groups appear to have higher total cholesterol levels, especially since 1998 [7].

Despite a plethora of information on the effectiveness of statins at the individual level, especially for secondary prevention, their contribution to the total cholesterol fall in the wider population remains unclear. Farzadfar et al. and Cohen et al. suggest that statins are important in lowering population mean total cholesterol in high income countries including the United States (US) [8,9]. However, it seems that this is neither completely true, nor universal because: 1) large falls in total cholesterol occurred before statins were widely used [10,11]; and 2) the large recent total cholesterol falls observed in Iceland, Sweden, Czech and Finland are principally attributed to improved diets [12–15]. In addition, there are policy concerns over statins and health inequalities. This is because statin prescription is a healthcare based intervention, requiring individual action, which might potentially increase inequalities [16,17].

The debate about statins for primary prevention of CVD has become heated. Last year, the American College of Cardiology (ACC) and the American Heart Association (AHA) updated their recommendations for the treatment of total cholesterol, substantially widening the criteria for statin prescription in otherwise healthy individuals [18]. Now, the UK National Institute for Health and Care Excellence (NICE) has made similar recommendations to drop the ten-year annual risk threshold from 20% to 10%, and almost double the number of eligible adults, from 7 million to 12 million [19]. This has proved very controversial [20,21].

The primary objective of this study was to quantify the contribution of statins to the observed fall in population mean cholesterol levels in England over the past two decades. A secondary objective was to look for any differences in this contribution between socioeconomic groups.

## Methods

We analysed changes in observed mean total cholesterol levels in the adult England population between 1991–92 (baseline) and 2011–12. We then compared the observed changes with a hypothetical counterfactual ‘no statins’ scenario, where the impact of statins on population total cholesterol was estimated and removed. Therefore, the ‘no statins’ scenario estimates the hypothetical mean cholesterol of the population, if statins were not available and the population had no benefit from them. Any gap between the observed and the estimated mean total cholesterol would then be attributed to all other possible drivers of population cholesterol levels, principally diet. We stratified our analysis by age-group, sex and, where possible and relevant, by quintiles of the 2010 Index of Multiple Deprivation (QIMD) [22].

### Survey data

Specifically, we used anonymised, non-identifiable, participant-level data from the Health Survey for England (HSE) for the two respective periods [23–25]. For the 2011–12 period we aggregated the data of HSE 2011 and HSE 2012, while for 1991–92 this was independently performed by HSE analysts. These cross-sectional surveys provide a representative sample of the non-institutionalised population in England for the respective years. The data files contained anonymised, individualised information for all the participants. We excluded participants younger than 18 years old. For HSE 2011–12 both the weighting and the sampling design were considered in the estimation of all the point estimates and their standard errors. In particular, the weighting adjusts both for selection and non-response bias. The sample for HSE 1991–92 was un-weighted, therefore, only the sampling design was taken into account. Further details about HSE can be found elsewhere [26–28].

### Socioeconomic stratification

There were no common socioeconomic indicators between the two samples; QIMD was therefore used for the 2011–12 sample and social class based on occupation (I—V) was used for the 1991–92 sample.

QIMD is a measure of relative area deprivation based on the 2010 version of the Index of Multiple Deprivation [22]. According to this system, all Lower Super Output Areas in England (LSOA) (average population of 1,500) are ranked in order of increasing deprivation, based on seven domains of deprivation: income; employment; health deprivation and disability; education, skills and training; barriers to housing and services; crime and disorder, and living environment. For the ranking, individual level information about the habitats of these areas is used from multiple sources. Then, the QIMD is formed from the quintiles of the above index, one through five, where quintile one is considered the ‘most affluent’ and quintile five the ‘most deprived’. The HSE team provided the QIMD of each participant for HSE 2011–12 based on their postcode of residence, which is a sub-division of LSOAs. We opted to use the QIMD instead of other available socioeconomic classification systems mainly for three reasons. First, the QIMD was the only socioeconomic indicator that had no missing cases in our data, second, for our results to be comparable with other studies that used QIMD and third, because QIMD is extensively used by local public health departments, Office of National Statistics and researchers in England.

The HSE 1991–92 social class classification was based on the 1990 version of the Standard Occupational Classification (SOC90) [29] and the self-reported occupation of the participants. Social class was provided as a variable in the data, by the HSE team. We aggregated full time students, armed forces personnel, those who never worked, and those whose occupation was not fully described in one category (‘Other’). In our analysis, we avoided any direct comparisons between the two socioeconomic classification systems.

### Total cholesterol measurement

Total cholesterol is reported in millimoles per litre (mmol/L). To convert it to milligrams per decilitre (mg/dL) please multiply the reported cholesterol values by 38.6. In 2011–12 a sub-sample of the total HSE sample was eligible and consented to provide non-fasting blood samples for the measurement of total cholesterol in serum. For HSE 1991–92, participants aged 18 and over were asked to provide a blood sample for the same purpose. Since April 2010 the equipment that was used for the measurement of total cholesterol for HSE was replaced. The effect of this change was that measured concentrations of total cholesterol from this date onwards were on average 0.1mmol/L higher. We adjusted for this difference in our analyses by subtracting 0.1mmol/L from the respective total cholesterol measurements. A more detailed description of the total cholesterol measurement process can be found elsewhere (pages 32–36 in [26], and pages 31–35 in [27]).

### Estimating statin utilisation

In England, individuals may have access to statins using two available routes. Statins can either be prescribed to them by a doctor (or a non-medical prescriber), or they can be bought over the counter (OTC) from a pharmacy with or without prior expert advice. HSE assessed both routes. In 2011–12, during a nurse interview, the participants were asked to report the medication that had been prescribed to them by a doctor or by a non-medical prescriber. Specifically for statins, they were also asked whether they bought OTC. Finally, those that had been prescribed a statin or bought it OTC were asked if they had used it during the past seven days. We only considered the participants that answered positively in the last question as statin users. For HSE 1991–92 the participants were asked similar questions during the nurse interview. However, statins were included in the wider category of lipid-lowering medication and were not prescribed for primary prevention [30,31]. Since the uptake of this category as a whole was very low, we assumed that statins had a negligible effect on total cholesterol at population level; thus, we ignored it completely (please see S1 Text for further justification of this assumption).

### Statistical analysis

The analysis was performed in R statistical software (v3.1.0) [32] including the R package “survey” [33]. An approximate 95% confidence interval (CI) for proportions (e.g. statin uptake) was calculated from the survey data using the incomplete beta function method, with an effective sample size based on the estimated variance of the proportion [34]. Missing cases were excluded from our analysis (please refer to Table 1).

**Table 1:** Samples baseline characteristics. Values are numbers (percentages).

	Number of participants interviewed by a nurse				Number of participants with a valid total cholesterol result			
	1991–92 (n = 7043)		2011–12 (n = 10965)		1991–92 (n = 4995)		2011–12 (n = 7772)	
Characteristics	Men	Women	Men	Women	Men	Women	Men	Women
**Age (years)**								
18–34	999 (14.2)	1165 (16.5)	877 (8.0)	1350 (12.3)	733 (14.7)	730 (14.6)	604 (7.8)	797 (10.3)
35–54	1148 (16.3)	1240 (17.6)	1632 (14.9)	2194 (20.0)	886 (17.7)	921 (18.4)	1216 (15.6)	1633 (21.0)
55+	1101 (15.6)	1390 (19.7)	2254 (19.7)	2658 (24.2)	806 (16.1)	919 (18.4)	1611 (20.7)	1911 (24.6)
**QIMD**								
1 (most affluent)	-	-	1058 (9.6)	1389 (12.7)	-	-	785 (10.1)	995 (12.8)
2	-	-	1057 (9.6)	1364 (12.4)	-	-	791 (10.2)	997 (12.8)
3	-	-	1017 (9.3)	1278 (11.7)	-	-	732 (9.4)	892 (11.5)
4	-	-	865 (7.9)	1133 (10.3)	-	-	606 (7.8)	781 (10.0)
5 (most deprived)	-	-	766 (7.0)	1038 (9.5)	-	-	517 (6.7)	676 (8.7)
**Social class**								
I Professional	235 (3.3)	53 (0.8)	-	-	174 (3.5)	41 (0.8)	-	-
II Managerial technical	908 (12.9)	856 (12.2)	-	-	688 (13.8)	610 (12.2)	-	-
IIIN Skilled non-manual	320 (4.5)	1304 (18.5)	-	-	238 (4.8)	909 (18.2)	-	-
IIIM Skilled manual	1085 (15.4)	388 (5.5)	-	-	816 (16.3)	251 (5.0)	-	-
IV Semi-skilled manual	460 (6.5)	693 (9.8)	-	-	343 (6.9)	464 (9.3)	-	-
V Unskilled manual	157 (2.2)	363 (5.2)	-	-	112 (2.2)	225 (4.5)	-	-
Other	83 (1.2)	138 (2.0)	-	-	54 (1.1)	70 (1.4)	-	-

The difference between the number of participants that had a nurse interview and those who had a valid total cholesterol result indicates the missing cases. QIMD denotes quintiles of index of multiple deprivation (1 = most affluent, 5 = most deprived).

To test the statistical significance of socioeconomic trends in total cholesterol, against the null hypothesis of ‘no trend’, we fitted a generalised linear model, with inverse-probability weighting and design-based standard errors. Specifically, we treated total cholesterol measurements as the dependent variable and the QIMD (or social class) as the independent one. We considered QIMD and social class as numeric variables for this (e.g. QIMD 1 through 5 represented the 5 quintiles and social class 1 through 7 represented the social classes I, II, IIIN, IIIM, IV, V and ‘Other’ respectively). Therefore, the *β* coefficient (slope) of the QIMD (or social class) and its standard error was a measure of the socioeconomic gradient. When *β* was not statistically significant we assumed no socioeconomic gradient. When *β* was statistically significant, its sign revealed the direction of the gradient (e.g. a negative sign means that mean total cholesterol is lower among the more deprived groups) and its absolute value measured the magnitude of the gradient.

A similar approach was followed to explore socioeconomic trends in statin utilisation. Since this time the dependent variable was a binary one, we used a binomial model.

#### Estimating the effect of statins

The average effect of each specific statin and strength on an individual’s total cholesterol is known from the literature [35–38]. However, the exact type of statin, and strength, had not been recorded for the participants in HSE 2011–12. To overcome this limitation we used the exact amount of statins (by proprietary name and strength) that were both prescribed and dispensed in England for 2011 and 2012, available from the Health and Social Care Information Centre [39,40]. We then estimated a weighted mean of the proportional decrease of total cholesterol attributable to statins overall (Eq 1).

$$E_{w}=\frac{\sum_{i}\sum_{j}\left(M_{ij}*E_{ij}\right)}{\sum_{i}\sum_{j}\left(M_{ij}\right)}$$ (1)

Eq 1. Formula for the estimation of the proportional decrease in mean total cholesterol attributable to overall statins use.

Where:

*E*_*w*_ is the proportional decrease in mean total cholesterol attributable to statins, among statin users

*E*_*ij*_ is the proportional decrease in mean total cholesterol attributable to a specific statin *i* of a specific strength *j* (e.g. Simvastatin 20mg)

*M*_*ij*_ is the number of units of a specific statin *i* and strength *j* that have been prescribed and dispensed. For liquid forms 5ml were considered as one unit, otherwise one tablet was considered as a unit

For the estimation of *E*_*ij*_ data from several meta-analysis were used as follows: We obtained the mean and standard error (calculated directly from the 95% CI assuming approximate normality) of the proportional reduction in serum low-density lipoprotein (LDL) from the meta-analysis of Law et al. [35]. The proportional reduction was derived from the absolute reduction, standardised to usual serum LDL of 4.8 mmol/L before treatment, and it was independent of the pre-treatment LDL. This allowed us to use a weighted mean approach on proportions. We then converted the LDL reduction into total cholesterol reduction using data from other studies, [36–38] assuming a linear relation between total cholesterol and LDL reduction. For strengths not included in the above meta-analysis (e.g. Atorvastatin 30mg), we used a linear regression model to estimate their effect, based on the effect of known strengths. Specifically, we treated the total cholesterol reduction as the dependent variable and the natural logarithm of strength as the independent one. We weighted the model against the inverse variance of the cholesterol reduction. The effectiveness of solid and liquid forms was considered equal. Similarly, the effectiveness of the combined forms of simvastatin with ezetimibe was considered equal to the effectiveness of same strength simvastatin (S1 Table). The standard error of *E*_*w*_ was estimated using the Cochran’s definition for the standard error of the weighted mean [41,42].

For the ‘no statins’ scenario, we calculated the predicted total cholesterol for each statin user, with the effect of statin removed using the formula below (Eq 2).

$$TC_{pred}=\frac{TC_{obs}}{1-E_{w}}$$ (2)

Eq 2. Formula for the calculation of predicted total cholesterol with the effect of statins removed.

Where:

*TC*_*pred*_ is the predicted total cholesterol of the statin user with the statin effect removed

*TC*_*obs*_ is the observed total cholesterol of the statin user

*E*_*w*_ is the proportional decrease in mean total cholesterol attributable to statins, derived from Eq 1.

We used Monte Carlo simulation to incorporate the uncertainty from the sampling distribution of *E*_*w*_. For each statin user we drew 1000 values from a normal distribution with mean *E*_*w*_ and standard deviation as per the estimated standard error (described above). We then averaged over the *TC*_*pred*_ predictions and considered this mean value as the predicted total cholesterol of each statin user, with the statin effect removed.

#### Quantifying the contribution of statins on population’s mean total cholesterol reduction

To quantify and compare the contribution of statins against the contribution of all other total cholesterol lowering interventions in the population, we first plotted the mean total cholesterol for 1991–92, 2011–12 and the ‘no statins scenario’ by age for each sex. We considered the area enclosed by the respective curves for 1991–92 and 2011–12 as representing the full observed cholesterol reduction (area A). Therefore, the area enclosed by the 2011–12 and the ‘no statin’ scenario represents the reduction of cholesterol attributable to statins (area B). Thus, the fraction (area B) / (area A) expresses the contribution of statins to the observed decline of mean total cholesterol. For the estimation of areas A and B we used natural spline interpolation as implemented in the R package “MESS” [43].

To estimate the uncertainty intervals (UI) around the estimated contribution of statins, we modified the previous method to allow for a Monte Carlo simulation approach. Specifically, for each age in the population, we drew 10000 values from the conditional sampling distribution, which we approximated by a normal distribution with age-specific estimate mean and standard error. These are then averaged across the age range to yield a point estimate, and 2.5% and 97.5% percentiles were used to define the 95% UI. Due to small representation of ages above 89 in our sample, we aggregated participants older than 89 years with those aged 89.

Finally, we repeated the analysis separately for each QIMD under the assumption that total cholesterol had no socioeconomic gradient in 1991–92. We further limited the analysis in participants younger than 76 years because of the small number of older participants in our sample, when stratified by QIMD. To test the statistical significance of any observed socioeconomic trend we used the two-tailed Cochran-Armitage trend test.

#### Sensitivity analysis

For the estimation of *E*_*w*_ several assumptions were involved that do not necessarily reflect on its estimated standard error. We repeated our analysis after we multiplied the standard error of *E*_*w*_ by a factor of 10 in order to test the robustness of our results with a higher than measured uncertainty scenario.

### Ethical approval

Ethical approval for the 2011 and 2012 surveys was obtained from the Oxford A Research Ethics Committee (reference numbers 10/H0604/56) by the Health Survey for England team. For 1991 and 1992 surveys ethical approval had been granted by the Local Research Ethics Councils in England. Anonymised, non-identifiable data of HSE are available to academics and public sector staff through the UK Data Archive (www.data-archive.ac.uk) for secondary analysis, without requiring further approval.

## Results

The baseline characteristics of the 1991–92 and 2011–12 samples are summarised in Table 1, while mean total cholesterol values by age group and sex are presented in Table 2 (1991–92) and Table 3 (2011–12). Overall, the prevalence of statin use in England, including OTC statin users was 13.2% (95% CI: 12.5% to 14.0%) in 2011–12. Another 0.8% (95% CI: 0.6% to 1.0%) of the population were prescribed or bought OTC statins; however, they did not use them for at least a week before the nurse interview.

**Table 2:** Observed mean total cholesterol (mmol/L) overall, and by age group, sex and social class in England, 1991–92.

	18–34 (years)		35–54		55+		
Social class	Men	Women	Men	Women	Men	Women	Overall
I Professional	5.52 (5.20 to 5.83)	5.10 (4.70 to 5.50)	5.95 (5.71 to 6.19)	5.64 (5.26 to 6.03)	5.99 (5.66 to 6.31)	6.62 (6.12 to 7.12)	5.64 (5.48 to 5.81)
II Managerial technical	5.25 (5.06 to 5.44)	5.05 (4.93 to 5.17)	6.01 (5.89 to 6.13)	5.57 (5.46 to 5.69)	6.24 (6.10 to 6.39)	6.79 (6.62 to 6.97)	5.69 (5.58 to 5.82)
IIIN Skilled non-manual	5.24 (5.06 to 5.43)	5.02 (4.92 to 5.12)	6.15 (5.88 to 6.41)	5.71 (5.58 to 5.83)	6.08 (5.81 to 6.36)	6.80 (6.66 to 6.94)	5.64 (5.49 to 5.79)
IIIM Skilled manual	5.16 (5.04 to 5.27)	5.05 (4.81 to 5.29)	5.93 (5.78 to 6.07)	5.97 (5.70 to 6.24)	6.06 (5.95 to 6.18)	6.83 (6.61 to 7.05)	5.72 (5.61 to 5.84)
IV Semi-skilled manual	5.16 (4.95 to 5.37)	5.12 (4.96 to 5.27)	5.89 (5.68 to 6.11)	5.70 (5.53 to 5.87)	6.00 (5.82 to 6.19)	6.95 (6.76 to 7.14)	5.70 (5.55 to 5.85)
V Unskilled manual	5.25 (4.82 to 5.68)	5.15 (4.84 to 5.45)	6.07 (5.67 to 6.47)	6.00 (5.77 to 6.22)	6.04 (5.63 to 6.45)	6.97 (6.54 to 7.41)	6.00 (5.79 to 6.21)
Other	4.70 (4.39 to 5.01)	5.14 (4.82 to 5.46)	5.82 (5.03 to 6.61)	5.03 (4.48 to 5.57)	6.37 (5.62 to 7.13)	6.70 (6.14 to 7.26)	5.27 (5.06 to 5.49)
**All**	5.20 (5.12 to 5.27)	5.06 (5.00 to 5.13)	5.97 (5.90 to 6.05)	5.70 (5.64 to 5.77)	6.10 (6.03 to 6.18)	6.84 (6.76 to 6.93)	
**Slope of the trend**	-0.07 (-0.13 to -0.01)	0.02 (-0.02 to 0.07)	-0.02 (-0.07 to 0.03)	0.05 (0.00 to 0.10)	-0.04 (-0.10 to 0.02)	0.04 (-0.04 to 0.11)	0.00 (-0.02 to 0.02)
***P* for trend**	**0.01**	0.27	0.47	**0.03**	0.19	0.32	0.96

Socioeconomic trends are also presented. Brackets contain 95% confidence intervals. The ‘Overall’ column is adjusted for age and sex.

**Table 3:** Observed mean total cholesterol (mmol/L) overall, and by age group, sex and quintiles of index of multiple deprivation (QIMD) (1 = most affluent, 5 = most deprived) in England, 2011–12.

	18–34 (years)		35–54		55+		
QIMD	Men	Women	Men	Women	Men	Women	Overall
1 (most affluent)	4.80 (4.60 to 5.00)	4.76 (4.60–4.92)	5.53 (5.42 to 5.64)	5.24 (5.13 to 5.36)	5.12 (5.01 to 5.23)	5.77 (5.67 to 5.87)	5.19 (5.09 to 5.29)
2	4.71 (4.56 to 4.86)	4.46 (4.31 to 4.61)	5.47 (5.33 to 5.61)	5.19 (5.08 to 5.31)	5.07 (4.95 to 5.19)	5.72 (5.61 to 5.82)	5.09 (4.99 to 5.20)
3	4.63 (4.41 to 4.86)	4.70 (4.53 to 4.87)	5.64 (5.50 to 5.79)	5.26 (5.15 to 5.38)	5.05 (4.91 to 5.18)	5.67 (5.54 to 5.80)	5.10 (4.99 to 5.22)
4	4.84 (4.65 to 5.02)	4.61 (4.46 to 4.77)	5.46 (5.30 to 5.62)	5.35 (5.20 to 5.49)	4.95 (4.80 to 5.11)	5.55 (5.40 to 5.70)	5.05 (4.94 to 5.17)
5 (most deprived)	4.79 (4.57 to 5.01)	4.59 (4.44 to 4.74)	5.40 (5.24 to 5.57)	5.31 (5.17 to 5.45)	4.74 (4.55 to 4.92)	5.34 (5.15 to 5.54)	4.93 (4.82 to 5.05)
**All**	4.75 (4.66 to 4.84)	4.62 (4.55 to 4.69)	5.50 (5.44 to 5.57)	5.26 (5.21 to 5.32)	5.02 (4.96 to 5.08)	5.64 (5.58 to 5.70)	
**Slope of the trend**	0.02 (-0.05 to 0.08)	-0.01 (-0.06 to 0.04)	-0.03 (-0.07 to 0.02)	0.03 (-0.01 to 0.07)	-0.08 (-0.12 to -0.03)	-0.10 (-0.14 to -0.05)	-0.03 (-0.05 to -0.01)
***P* for trend**	0.60	0.67	0.26	0.16	**<0.001**	**<0.001**	**0.002**

Socioeconomic trends are also presented. The ‘Overall’ column is adjusted for age and sex. Brackets contain 95% confidence intervals.

For 1991–92, statin use was not specifically recorded in the survey; however, the prevalence of all lipid lowering medications, including statins, was 0.5% (95% CI: 0.3% to 1.0%). Table 4 summarises the prevalence of statin use in England for 2011–12 by age group, sex and QIMD. There was a statistically significant socioeconomic gradient in ages above 35 years for both sexes, where the use of statins increased with deprivation.

**Table 4:** Prevalence of statin use in England 2011–12 by age, sex and quintiles of index of multiple deprivation (QIMD) (1 = most affluent, 5 = most deprived).

	18–34 (years)		35–54		55+		
QIMD	Men	Women	Men	Women	Men	Women	Overall
1 (most affluent)	0% (0–2%)	-	5% (3–8%)	2% (1–3%)	36% (32–41%)	20% (16–23%)	19% (16–23%)
2	-	-	7% (4–11%)	3% (2–5%)	38% (34–43%)	24% (20–27%)	22% (18–26%)
3	0% (0–2%)	0% (0–2%)	7% (5–11%)	2% (1–4%)	32% (28–37%)	29% (25–33%)	20% (16–24%)
4	1% (0–5%)	-	8% (5–12%)	4% (2–6%)	39% (34–44%)	29% (25–34%)	20% (17–24%)
5 (most deprived)	-	1% (0–3%)	9% (6–13%)	8% (5–11%)	47% (40–54%)	34% (29–40%)	21% (17–25%)
**All**	0% (0–1%)	0% (0–1%)	7% (6–9%)	4% (3–4%)	38% (36–40%)	26% (25–28%)	
***P* for trend**	-	-	**0.03**	**< 0.001**	**0.04**	**<0.001**	**<0.001**

The ‘Overall’ column is adjusted for age and sex. Brackets contain 95% confidence intervals.

In 2011–12, some 13.1% (95% CI: 12.4 to 14.0%) of study population used statins prescribed to them (not including OTC users), over the seven days before the survey interview. We estimated the expected number of units (e.g. tablets or 5ml doses of liquid statins) that were consumed in England for the same period, assuming that they stayed on statins for the whole year and that institutionalised population shares the same consumption attitudes, to be approximately 4.00 billion. This showed reassuringly close agreement with the observed unit consumption of almost 4.07 billion [39,40], being just 1.5% lower.

**Fig 1:**
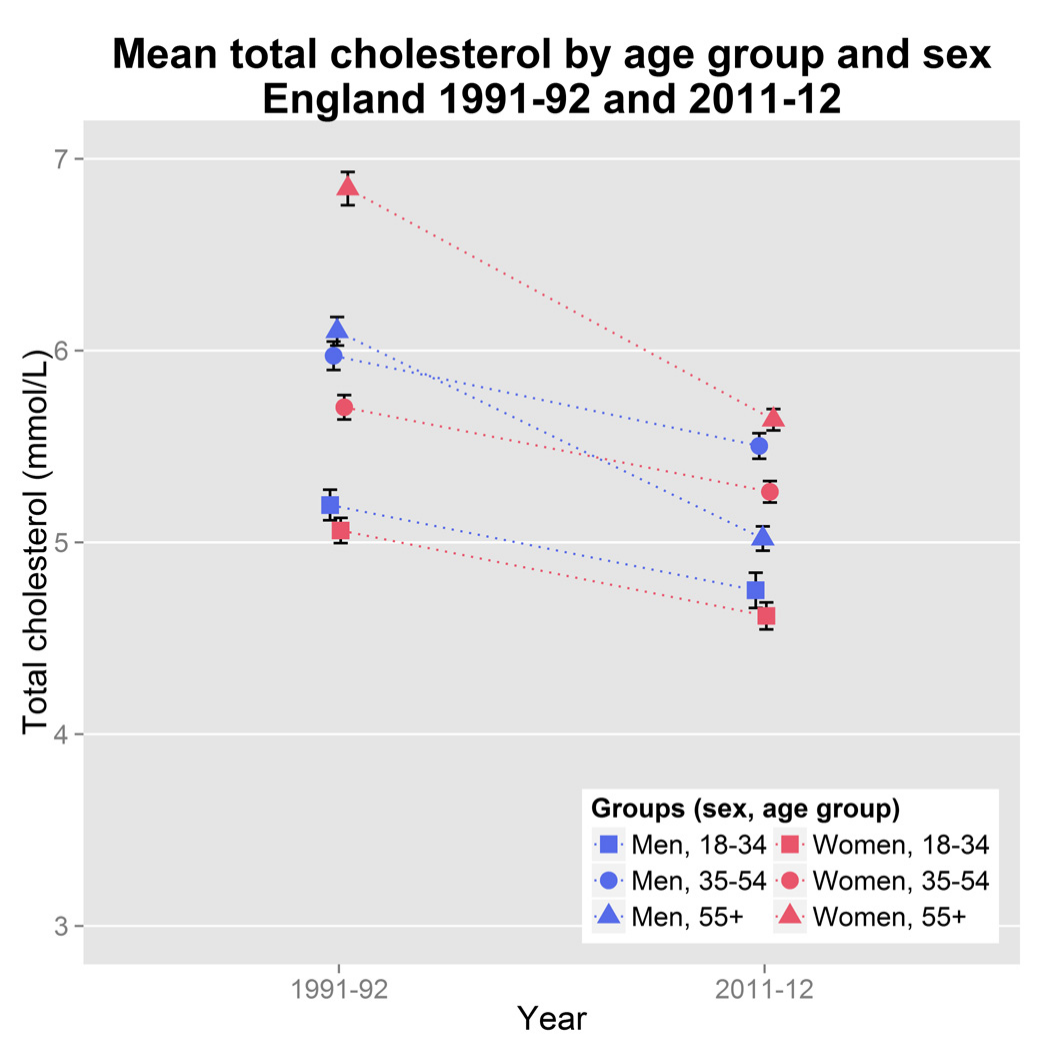
Mean serum total cholesterol (mmol/L) observed decline in England from 1991–92 to 2011–12 in men and women by age group. The error bars depict 95% confidence interval of the means. The vertical axis starts at 3 mmol/L to improve readability. The dotted lines are visual aids and do not reflect linear fits.

The mean total cholesterol of adult non-institutionalised population in England decreased from 5.86 mmol/L (95% CI: 5.82 to 5.90) in 1991–92 to 5.17 mmol/L (95% CI: 5.14 to 5.20) in 2011–12. The decrease was observed in all age groups and it was steeper for ages over 55 for women and 35 for men (Fig 1). The inverse socioeconomic gradient observed since 1998 [7] persisted overall and in the subgroup of those aged over 55 years. No gradient was observed for other age groups (Table 3). On the contrary, we did not observe any socioeconomic gradient in 1991–92 with social class as a socioeconomic indicator when adjusted for age and sex (Table 2). The trend remained non-significant even when we placed the ‘Other’ social class group before all other groups.

### ‘No statins’ scenario

We estimated the total effect of statins on total cholesterol reduction using Eq 1 as *E*_*w*_ = 25.7% (95% CI: 23.3% to 28.0%). The mean predicted total cholesterol *TC*_*pred*_ of the population was calculated to be 5.36 mmol/L (95% CI: 5.33 to 5.40).

**Fig 2:**
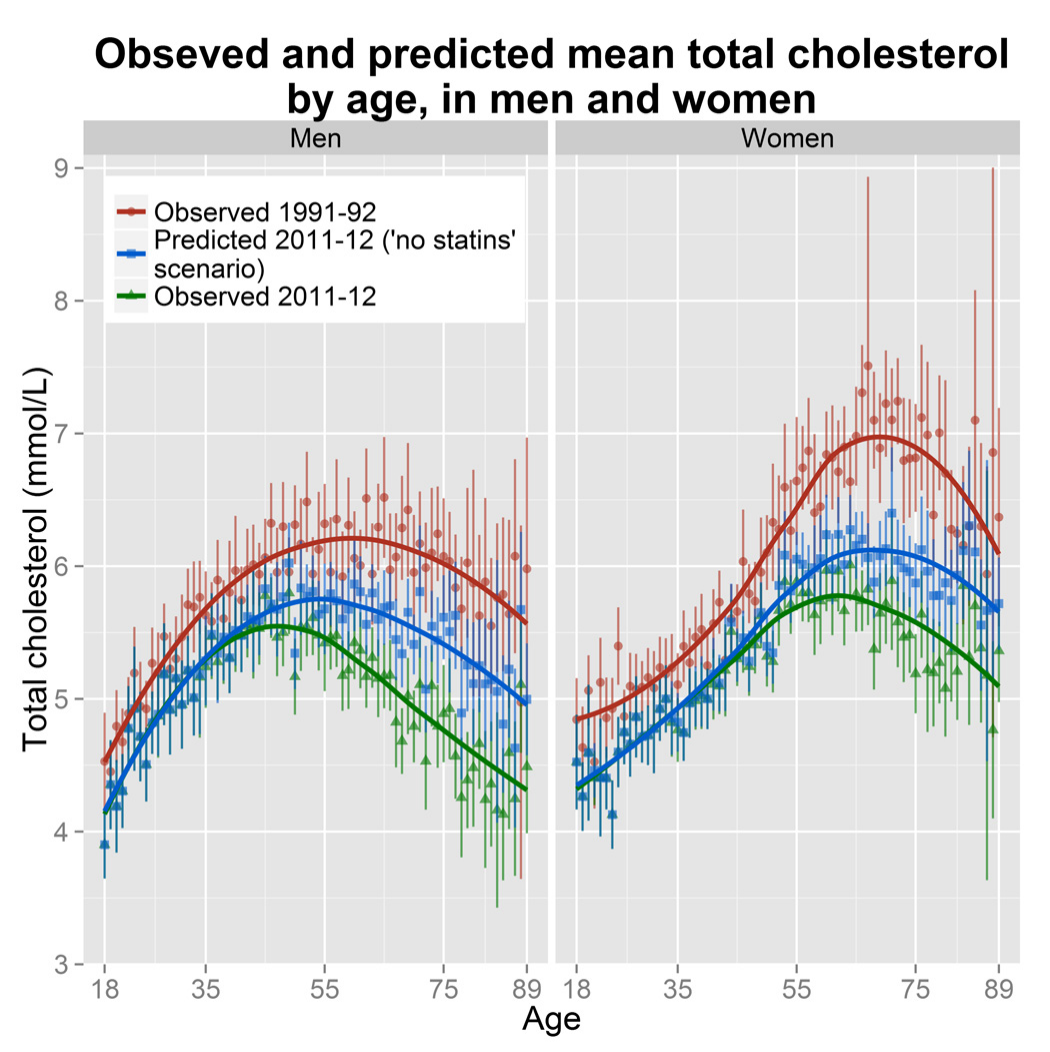
Mean serum total cholesterol by age, in men and women, in England (observed and predicted values). The points depict the mean total cholesterol and the vertical lines 95% confidence intervals (CI). The curves were derived from weighted local regressions and are used to enhance readability. Due to small sample sizes we aggregated participants aged 89 with those older than 89 years. To improve readability the axes are not numbered from 0.

Fig 2 depicts the predicted mean total cholesterol of the population without the effect of statins, against the observed mean total cholesterol in 1991–92 and 2011–12, by age and sex. When the effect of statins was removed, the inverse socioeconomic gradient of cholesterol in the overall population disappeared (slope -0.01, 95% CI: -0.03 to 0.01, *P* = 0.45). Subgroup analysis revealed that for men over 55 the slope was reduced to -0.05 (95% CI: -0.10 to -0.01, *P = 0*.*03*) and for women over 55 the gradient was essentially zero (slope -0.04, 95% CI: -0.08 to 0.01, *P =* 0.09). In addition, a socioeconomic trend appeared for women between 35 and 54 years with a slope of 0.05 (95% CI: 0.01 to 0.10, *P =* 0.01). We saw no other statistically significant gradient, for the remaining age groups (S2 Table).

Finally, statins were estimated as responsible for approximately 33.7% (95% UI: 28.9% to 38.8%) of the total cholesterol reduction since 1991–92. When stratified by sex statins contribution was 40.1% (95% UI: 33.6% to 47.7%) in men and 28.6% (95% UI: 22.3% to 35.0%) in women. Table 5 summarises the contribution of statins for each socioeconomic group, by age group and sex. The negative values in the UI, implying that statins could have increased cholesterol to some, are an artefact of the Monte Carlo simulation due to wide mean cholesterol CI overlapping in some ages. Statins’ contribution was consistently higher among men, consistent with the observed higher utilisation.

**Table 5:** Estimated proportional contribution of statins to total cholesterol reduction since 1991–92 for each quintile of index of multiple deprivation (QIMD), by age group and sex.

	35–54 (years)		55–75		18–75	
QIMD	Men	Women	Men	Women	Men	Women
1 (most affluent)	14.0% (-19.2 to 41.9%)	4.2% (-24.4 to 28.3%)	50.6% (36.2 to 64.6%)	24.4% (11.4 to 36.6%)	33.5% (15.6 to 49.9%)	15.9% (1.9 to 28.9%)
2	13.0% (-28.2 to 45.9%)	5.9% (-23.9 to 30.7%)	59.7% (43.5 to 75.7%)	23.9% (10.2 to 36.8%)	36.0% (19.5 to 51.2%)	14.3% (2.0 to 25.5%)
3	17.9% (-49.7 to 78.2%)	3.6% (-33.9 to 33.3%)	37.8% (21.3 to 52.7%)	36.0% (21.2 to 50.1%)	26.9% (7.3 to 44.6%)	23.5% (7.5 to 37.5%)
4	29.0% (-7.2 to 58.9%)	19.3% (-37.9 to 64.4)	45.0% (27.7 to 60.6%)	36.3% (21.3 to 50.5%)	34.4% (19.0 to 48.9%)	24.8% (9.2 to 38.9%)
5 (most deprived)	31.1% (-9.1 to 63.8%)	37.1% (-19.0 to 79.9%)	43.2% (28.6 to 57.1%)	32.6% (16.0 to 47.9%)	33.8% (19.7 to 46.1%)	33.4% (18.3 to 47.5%)
**All**	22.2% (4.8 to 39.8%)	11.9% (-4 to 26.1%)	48.0% (40.1 to 56.1%)	40.0% (23.3 to 54.9%)	33.2% (25.8 to 40.6%)	21.3% (14.8 to 28.0%
***P* for trend**	0.24	**0.03**	0.41	0.17	0.99	**0.02**

Age group 18–34 was omitted as statins’ contribution was practically zero. Analysis was restricted to ages younger than 76 due to low number of older participants. Brackets contain 95% uncertainty intervals estimated by Monte Carlo.

### Sensitivity analysis

The mean predicted total cholesterol (*TC*_*pred*_) of the population, using the inflated standard error of *E*_*w*_, was calculated to be 5.39 mmol/L (95% CI: 5.35 to 5.42). This is less than a 0.03 mmol/L difference from the main analysis. For the subgroup of deprived men older than 55, with the highest statin utilisation, the *TC*_*pred*_ from the sensitivity analysis was 0.09 mmol/L higher than the one from the main analysis. Similarly, the contribution of statins to the observed cholesterol decline for the whole population was estimated to be 33.9% (95% UI: 28.8 to 38.7%), a 0.2% difference from the main analysis result. A similar pattern of minimal changes was observed for the remaining results.

## Discussion

This is the first study we know of to quantify the contribution of statins to the observed decrease of total cholesterol in England’s population by socioeconomic group. Our results strongly suggest that the statins were not the main driver of total cholesterol reduction since 1991–92. In fact, only around one third of the overall reduction might be attributed to statins, and that was mainly in patients aged over 55 years. Statins were more widely used in deprived than affluent areas. They appeared to help reduce socioeconomic inequalities in total cholesterol among women, but not among men.

### Statins utilisation

In our study, statins’ utilisation was higher in more deprived areas for men and women aged over 35 years. This socio-economic pattern may partly reflect the higher prevalence of CVD in more deprived areas [44] and the incentivised use of the QRISK score for cardiovascular risk stratification in clinics, which includes area deprivation as a risk factor [45,46]. Our findings are consistent with earlier studies that used different methodologies. Ashworth et al. and Wu et al. also found that statin prescription was higher in more deprived areas in the UK [47,48]. This success in tackling inequalities might be attributed to the National Health Service (NHS), since evidence from Australia, Sweden, Denmark and the US [49–52] suggest that statin prescription in these countries has a socioeconomic gradient, with a less than expected utilisation among the more disadvantaged, and potentially increases health inequalities.

### Statins contribution to cholesterol decline

The second interesting finding is the contribution of statins to the observed decline of total cholesterol since 1991–92. We found that statins are not the main driver of the cholesterol decline in England, echoing studies from Iceland, Sweden, Finland and the Czech Republic [12–15]. We estimated that only about a third of the observed total cholesterol decline could be attributed to statins. This contribution was slightly higher than the aforementioned studies, perhaps reflecting a more recent time period with correspondingly higher statin use in England 2011–12, and possible nuanced differences in methodologies. While the cholesterol decrease was observed in all age groups since 1991, statins mostly contributed to the fall in people older than 55 years.

The observed inverse socioeconomic gradient in total cholesterol levels might be partly attributed to statins. In the ‘no statins’ scenario the gradient disappeared completely when all ages were considered. However, the statin contribution varied across different genders and socioeconomic groups. Statin utilisation was higher in the most deprived groups, but inequitable by gender, reaching barely one third in women (34%) but almost half (47%) of deprived men in the 55+ age group. This difference can only partly be explained by the higher CVD prevalence among men. By contrast, the statin contribution to cholesterol lowering was rather stable across socio-economic groups in men (some 33%), but rose from 16% to 33% in women. This suggests that the component of all other cholesterol reduction drivers had a higher impact among the most deprived men, while their effect among women of all socioeconomic background was more or less equal. This demands further research.

### Public health implications

Overall, our research supports the principle of statins being the second best option for primary prevention. Non-statin interventions account for two thirds of the total cholesterol reduction observed since 1991–92, which can be mostly attributed to dietary changes because physical activity levels have not increased substantially over this period [30,53] and the contribution of other factors affecting lipids is small and remained more or less stable. Indeed, United Nations Food and Agriculture Organization data indicate that the animal fat supply per capita in the UK has fallen by almost 25% since 1991 [54]. This echoes Rose’s original assertion that the greatest public health impact will be achieved through population-wide reductions in CVD risk than through interventions targeting high-risk individuals [55].

Furthermore, the recent proposed widening of criteria for statin prescription in primary prevention by the ACC/AHA [18] and NICE [19] has been questioned on grounds of effectiveness, cost-effectiveness, acceptability and safety [21]. These measures may prove to be less effective than anticipated because of cumulative attrition factors. Approximately half of the UK patients that are commenced on lipid lowering medication for primary prevention are ineligible according to the respective guidelines, while many eligible patients remain untreated [48]. Moreover, over half the patients commenced on statins for primary prevention have discontinued them within 1–2 years [56–59]. In addition to medicalising otherwise healthy individuals, some patients may also be tempted to adopt more unhealthy diets because of the false ‘reassurance’ that statins will compensate for the unhealthy behaviours [60]. Along with the increased resource requirements, an additional opportunity cost comes from undermining the primary driver of cholesterol decline—nutritional improvements at individual and national policy levels [61].

Regarding inequalities in health and inequities in care: our research suggests that English statin prescribing might be equitable. This represents a success for the socialised medicine provided by the NHS England. In contrast, statin-based cholesterol reduction was not equitable among men, being similar in the more affluent and more deprived groups. These results are intriguing, because healthcare-based interventions generally increase the inequality gap [16,17].

### Strengths and limitations

This study was grounded on the best available evidence to explore the research question. We integrated all the available data from HSE, a cross-sectional survey of very high quality, the Prescription Cost Analysis report, an accurate and precise report about prescriptions in England, and published meta-analyses on the effect of statins. The modelling approach allowed for the best use of all the available information. In fact, despite the assumptions regarding the effects of statins our results were robust to the sensitivity analysis. Any biases and errors were diluted because they only applied to the about 13% of the sample who were statin users.

However, our study has several limitations. First, it is based on self-reported statin prescription and adherence, and does not account for statin indications; however, consistent data from prescription cost analysis reports for 2011–12 [39,40] suggest that our estimated prevalence of statin-use is fairly accurate. Second, unlike HSE 2011–12, HSE 1991–92 was not weighted to adjust for non-response bias. Furthermore, no other HSE has recorded statin use separately from other lipid-lowering medication; this renders an interim point analysis between 1991 and 2011 practically impossible.

Third, there were no common or directly compatible socioeconomic indicators between the two surveys to allow for more accurate comparisons. Our assumption that there was no socioeconomic gradient of mean total cholesterol in 1991–92 is supported by our finding of no such gradient by social class in HSE 1991–92. This is consistent with Scholes et al. who also showed no socioeconomic gradient in 1994 using QIMD as socioeconomic indicator [7]. The Whitehall II cohort also showed no socioeconomic gradient for total cholesterol in 1985–88 [62]. Neither did our analysis consider other inequalities, for instance, ethnic minorities or people with mental health or illiteracy problems [47,63,64].

Fourth, the estimate of the statin effect *E*_*w*_ was derived mostly from short-term trials lasting less than one year. However, Edward et al. have shown that the statins effect remains fairly stable in trials lasting more than one year (Additional file 5 in [37]). In addition, the estimation of *E*_*w*_ assumes that the differences between each trial population and our study sub-population of statin users were the same for each statin.

Fifth, this analysis cannot fully control for other factors that interfere with lipid profiles and their prevalence in the population changed substantially over the last two decades. BMI and diabetes mellitus are possibly the most important of them.

Finally, we used the statins effects reported in clinical trials, acknowledging that this might overestimate the real world efficacy of these drugs (mostly because of selection bias in the trials and reduced compliance in the population). However, this result in an overestimation of the contribution of statins, and thus its real contribution might have been even less than one third.

## Conclusions

Our research suggests that statins contributed about one third of the observed total cholesterol decline in England since 1991–92, and that their impact on reducing socioeconomic inequalities in total cholesterol was generally positive. However, the proposed wider indications for statins in primary prevention remains contested.

Further research is now needed to quantify the potential contribution of primary prevention statins to the ‘hard’ outcomes of cardiovascular morbidity and mortality in the UK. There is sufficient current evidence, however, to justify reconsidering the priorities of different interventions for the primary prevention of CVD.

## Supporting Information

S1 Table Statins effects and weights used for the estimation of the weighted mean E_w_. (DOCX)

S2 Table Predicted mean total cholesterol (mmol/L) with the statins effect removed. Overall, and by age group, sex and quintiles of index of multiple deprivation (QIMD) (1 = most affluent, 5 = most deprived) in England, 2011–12. Socioeconomic trends are also presented. Brackets contain 95% confidence intervals.
(DOCX)

S1 Text Supporting the assumption of no statin effect in 1991–1992. (DOCX)

## References

[1] Alwan A, Armstrong T, Bettcher D, Branca F, Chisholm D, Ezzati M, et al. (2011) Global status report on noncommunicable diseases 2010. World Health Organization. Available: http://whqlibdoc.who.int/publications/2011/9789240686458_eng.pdf.

[2] Townsend N, Wickramasinghe K, Bhatnagar P, Smolina K, Nichols M, Leal J, et al. (2012) Coronary heart disease statistics 2012 edition. British Heart Foundation. Available: http://www.bhf.org.uk/publications/view-publication.aspx?ps=1002097.

[3] Unal B, Critchley JA, Capewell S (2004) Explaining the decline in coronary heart disease mortality in England and Wales between 1981 and 2000. Circulation 109: 1101–1107. 10.1161/01.CIR.0000118498.35499.B2 14993137

[4] The Global Burden of Metabolic Risk Factors of Chronic Diseases Collaborating Group (n.d.) Global Burden of Metabolic Risk Factors of Chronic Diseases. Available: http://www1.imperial.ac.uk/publichealth/departments/ebs/projects/eresh/majidezzati/healthmetrics/metabolicriskfactors/. Accessed 6 October 2014.

[5] Morton GM, Lee SM, Buss DH, Lawrance P (1995) Intakes and major dietary sources of cholesterol and phytosterols in the British diet. J Hum Nutr Diet 8: 429–440. 10.1111/j.1365-277X.1995.tb00338.x

[6] Department of Health (n.d.) Pharmacies and prescriptions: statistics. Available: http://webarchive.nationalarchives.gov.uk/20120503222906/http:/www.dh.gov.uk/en/PublicationsAndStatistics/Statistics/StatisticalWorkAreas/StatisticalHealthCare/DH_4086488. Accessed 15 June 2014.

[7] Scholes S, Bajekal M, Love H, Hawkins N, Raine R, O'Flaherty M, et al (2012) Persistent socioeconomic inequalities in cardiovascular risk factors in England over 1994–2008: A time-trend analysis of repeated cross-sectional data. BMC Public Health 12: 129 10.1186/1471-2458-12-129 22333887 PMC3342910

[8] Farzadfar F, Finucane MM, Danaei G, Pelizzari PM, Cowan MJ, Paciorek CJ, et al (2011) National, regional, and global trends in serum total cholesterol since 1980: systematic analysis of health examination surveys and epidemiological studies with 321 country-years and 3·0 million participants. The Lancet 377: 578–586. 10.1016/S0140-6736(10)62038-7 21295847

[9] Cohen JD, Cziraky MJ, Cai Q, Wallace A, Wasser T, Crouse JR, et al (2010) 30-year trends in serum lipids among United States adults: results from the National Health and Nutrition Examination Surveys II, III, and 1999–2006. Am J Cardiol 106: 969–975. 10.1016/j.amjcard.2010.05.030 20854959

[10] Pietinen P, Vartiainen E, Seppänen R, Aro A, Puska P (1996) Changes in diet in Finland from 1972 to 1992: impact on coronary heart disease risk. Prev Med 25: 243–250. 10.1006/pmed.1996.0053 8781001

[11] Johnson CL, Rifkind BM, Sempos CT, Carroll MD, Bachorik PS, Briefel RR, et al (1993) Declining serum total cholesterol levels among US adults: The National Health and Nutrition Examination Surveys. JAMA 269: 3002–3008. 10.1001/jama.1993.03500230084034 8501842

[12] Thorsson B, Steingrimsdottir L, Halldorsdottir S, Andersen K, Sigurdsson G, Aspelund T, et al (2013) Changes in total cholesterol levels in Western societies are not related to statin, but rather dietary factors: the example of the Icelandic population. Eur Heart J 34: 1778–1782. 10.1093/eurheartj/ehs395 23209261

[13] Eliasson M, Janlert U, Jansson JH, Stegmayr B (2006) Time trends in population cholesterol levels 1986–2004: influence of lipid-lowering drugs, obesity, smoking and educational level. The northern Sweden MONICA study. J Intern Med 260: 551–559. 10.1111/j.1365-2796.2006.01730.x 17116006

[14] Cífková R, Škodová Z, Bruthans J, Adámková V, Jozífová M, Galovcová M, et al (2010) Longitudinal trends in major cardiovascular risk factors in the Czech population between 1985 and 2007/8. Czech MONICA and Czech post-MONICA. Atherosclerosis 211: 676–681. 10.1016/j.atherosclerosis.2010.04.007 20471016

[15] Valsta LM, Tapanainen H, Sundvall J, Laatikainen T, Männistö S, Pietinen P, et al (2010) Explaining the 25-year decline of serum cholesterol by dietary changes and use of lipid-lowering medication in Finland. Public Health Nutr 13: 932–938. 10.1017/S1368980010001126 20513263

[16] Blackman T (2007) Statins, saving lives, and shibboleths. BMJ 334: 902 10.1136/bmj.39163.563519.55 17463464 PMC1857769

[17] Capewell S, Graham H (2010) Will cardiovascular disease prevention widen health inequalities? PLoS Med 7: e1000320 10.1371/journal.pmed.1000320 20811492 PMC2927551

[18] Stone NJ, Robinson JG, Lichtenstein AH, Bairey Merz CN, Blum CB, Eckel RH, et al (2014) 2013 ACC/AHA guideline on the treatment of blood cholesterol to reduce atherosclerotic cardiovascular risk in adults: a report of the American College of Cardiology/American Heart Association Task Force on Practice Guidelines. Circulation 129: S1–S45. 10.1161/01.cir.0000437738.63853.7a 24222016

[19] National Institute for Health and Care Excellence (2014) Lipid modification: cardiovascular risk assessment and the modification of blood lipids for the primary and secondary prevention of cardiovascular disease. NICE guidelines [CG181]. Available: http://www.nice.org.uk/Guidance/CG181. Accessed 26 August 2014. 25340243

[20] Wise J (2014) Open letter raises concerns about NICE guidance on statins. BMJ 348: 3937 10.1136/bmj.g3937 24920699

[21] Thompson R, Gerada C, Haslam D, Bamrah JS, Kendrick M, Malhotra A, et al. (2014) NICE statin letter: concerns about the latest NICE draft guidance on statins. Available: www.nice.org.uk/media/877/AC/NICE_statin_letter.pdf.

[22] Department for Communities and Local Government (2011) English indices of deprivation 2010—Publications—GOV.UK. Available: https://www.gov.uk/government/statistics/english-indices-of-deprivation-2010. Accessed 26 August 2014.

[23] NatCen Social Research, University College London. Department of Epidemiology and Public Health (2014) Health Survey for England, 2012 [computer file]. Colchester, Essex: UK Data Archive [distributor]. Available: 10.5255/UKDA-SN-7480-1. Accessed 1 May 2014.

[24] NatCen Social Research, University College London. Department of Epidemiology and Public Health (2013) Health Survey for England, 2011 [computer file]. Colchester, Essex: UK Data Archive [distributor]. Available: 10.5255/UKDA-SN-7260-1. Accessed 1 May 2014.

[25] Office of Population Censuses and Surveys. Social Survey Division (1997) Health Survey for England, 1991–1992: combined data file [computer file]. 2nd edition. Colchester, Essex: UK Data Archive [distributor]. Available: 10.5255/UKDA-SN-3238-1. Accessed 1 May 2014.

[26] Bridges S, Doyle M, Fuller E, Knott C, Mindell J, Moody A, et al. (2013) Health Survey for England 2012. Methods and documentation. Health and Social Care Information Centre. Available: http://www.hscic.gov.uk/catalogue/PUB13218/HSE2012-Methods-and-docs.pdf.

[27] Boniface S, Bridges S, Craig R, Darton R, Fuller E, Hancock R, et al. (2012) Health Survey for England 2011. Methods and documentation. Health and Social Care Information Centre. Available: http://www.hscic.gov.uk/catalogue/PUB09300/HSE2011-Methods-and-docs.pdf.

[28] UK Data Service (n.d.) Health Survey for England, 1991–1992: combined data file, documentation. Available: http://discover.ukdataservice.ac.uk/catalogue/?sn=3238&type=Data%20catalogue. Accessed 16 June 2014.

[29] Office for National Statistics (2012) Standard Occupational Classification and Socio-Economic Classification: Archive. Available: http://www.ons.gov.uk/ons/guide-method/classifications/archived-standard-classifications/soc-and-sec-archive/index.html. Accessed 27 August 2014.

[30] Capewell S, Ford ES (2011) Why have total cholesterol levels declined in most developed countries? BMC Public Health 11: 641 10.1186/1471-2458-11-641 21834954 PMC3199603

[31] Capewell S, Morrison CE, McMurray JJ (1999) Contribution of modern cardiovascular treatment and risk factor changes to the decline in coronary heart disease mortality in Scotland between 1975 and 1994. Heart 81: 380–386. 10.1136/hrt.81.4.380 10092564 PMC1729021

[32] R Core Team (2014) R: A Language and Environment for Statistical Computing. R Foundation for Statistical Computing. Available: http://www.R-project.org/.

[33] Lumley T (2013) Survey: analysis of complex survey samples. Available: http://cran.r-project.org/web/packages/survey/index.html.

[34] Korn LE, Graubard IB (1998) Confidence Intervals for Proportions with Small Expected Number of Positive Counts Estimated from Survey Data. Surv Methodol 24: 193–201.

[35] Law MR, Wald NJ, Rudnicka AR (2003) Quantifying effect of statins on low density lipoprotein cholesterol, ischaemic heart disease, and stroke: systematic review and meta-analysis. BMJ 326: 1423 10.1136/bmj.326.7404.1423 12829554 PMC162260

[36] Kong SX, Crawford SY, Gandhi SK, Seeger JD, Schumock GT, Lam NP, et al (1997) Efficacy of 3-hydroxy-3-methylglutaryl coenzyme A reductase inhibitors in the treatment of patients with hypercholesterolemia: a meta-analysis of clinical trials. Clin Ther 19: 778–797. 10.1016/S0149-2918(97)80102-6 9377621

[37] Edwards JE, Moore RA (2003) Statins in hypercholesterolaemia: A dose-specific meta-analysis of lipid changes in randomised, double blind trials. BMC Fam Pract 4: 18 10.1186/1471-2296-4-18 14969594 PMC317299

[38] Jones MD P, Kafonek MD S, Laurora PharmD I, Hunninghake MD D (1998) Comparative dose efficacy study of atorvastatin versus simvastatin, pravastatin, lovastatin, and fluvastatin in patients with hypercholesterolemia (the CURVES study). Am J Cardiol 81: 582–587. 10.1016/S0002-9149(97)00965-X 9514454

[39] Health and Social Care Information Centre (2013) Prescription Cost Analysis 2012. Available: http://www.hscic.gov.uk/searchcatalogue?productid=11412&q=prescription+cost+analysis&topics=0%2fPrescribing&sort=Relevance&size=10&page=1#top. Accessed 23 February 2014.

[40] Health and Social Care Information Centre 1 Trevelyan Square (2012) Prescription Cost Analysis 2011. Available: http://www.hscic.gov.uk/pubs/prescostanalysis2011. Accessed 23 February 2014.

[41] Cochran WG (1977) Sampling Techniques. 3rd edition New York: John Wiley & Sons 428 p.

[42] Gatz DF, Smith L (1995) The standard error of a weighted mean concentration—I. Bootstrapping vs other methods. Atmos Environ 29: 1185–1193. 10.1016/1352-2310(94)00210-C

[43] Ekstrom C (2014) MESS: Miscellaneous esoteric statistical script. Available: http://CRAN.R-project.org/package=MESS.

[44] Bajekal M, Scholes S, Love H, Hawkins N, O’Flaherty M, Raine R, et al (2012) Analysing recent socioeconomic trends in coronary heart disease mortality in England, 2000–2007: a population modelling study. PLoS Med 9: e1001237 10.1371/journal.pmed.1001237 22719232 PMC3373639

[45] Hippisley-Cox J, Coupland C, Vinogradova Y, Robson J, May M, Brindle P (2007) Derivation and validation of QRISK, a new cardiovascular disease risk score for the United Kingdom: prospective open cohort study. BMJ 335: 136 10.1136/bmj.39261.471806.55 17615182 PMC1925200

[46] Collins GS, Altman DG (2012) Predicting the 10 year risk of cardiovascular disease in the United Kingdom: independent and external validation of an updated version of QRISK2. BMJ 344: e4181–e4181. 10.1136/bmj.e4181 22723603 PMC3380799

[47] Ashworth M, Lloyd D, Smith RS, Wagner A, Rowlands G (2007) Social deprivation and statin prescribing: a cross-sectional analysis using data from the new UK general practitioner “Quality and Outcomes Framework.” J Public Health 29: 40–47. 10.1093/pubmed/fdl068 17071815

[48] Wu J, Zhu S, Yao GL, Mohammed MA, Marshall T (2013) Patient factors influencing the prescribing of lipid lowering drugs for primary prevention of cardiovascular disease in UK general practice: a national retrospective cohort study. PLoS ONE 8: e67611 10.1371/journal.pone.0067611 23922649 PMC3724846

[49] Stocks NP, Ryan P, McElroy H, Allan J (2004) Statin prescribing in Australia: socioeconomic and sex differences. A cross-sectional study. Med J Aust 180: 229–231. 14984343

[50] Ohlsson H, Lynch K, Merlo J (2010) Is the physician’s adherence to prescription guidelines associated with the patient’s socio-economic position? An analysis of statin prescription in South Sweden. J Epidemiol Community Health 64: 678–683. 10.1136/jech.2008.081166 19692716

[51] Thomsen RW, Johnsen SP, Olesen AV, Mortensen JT, Bøggild H, Olsen J, et al (2005) Socioeconomic gradient in use of statins among Danish patients: population-based cross-sectional study. Br J Clin Pharmacol 60: 534–542. 10.1111/j.1365-2125.2005.02494.x 16236044 PMC1884943

[52] Franks P, Tancredi D, Winters P, Fiscella K (2010) Cholesterol treatment with statins: Who is left out and who makes it to goal? BMC Health Serv Res 10: 68 10.1186/1472-6963-10-68 20236527 PMC2846927

[53] Shekelle RB, Shryock AM, Paul O, Lepper M, Stamler J, Liu S, et al (1981) Diet, serum cholesterol, and death from coronary heart disease: the Western Electric study. N Engl J Med 304: 65–70. 10.1056/NEJM198101083040201 7442730

[54] Food and Agriculture Organization of the United Nations (2014) FAOSTAT. Available: http://faostat.fao.org/default.aspx. Accessed 16 June 2014.

[55] Rose G (2001) Sick individuals and sick populations. Int J Epidemiol 30: 427–432. 10.1093/ije/30.3.427 11416056

[56] Ellis JJ, Erickson SR, Stevenson JG, Bemstein SJ, Stiles RA, Fendrick MA (2004) Suboptimal statin adherence and discontinuation in primary and secondary prevention populations. J Gen Intern Med 19: 638–645. 10.1111/j.1525-1497.2004.30516.x 15209602 PMC1492382

[57] Chaudhry HJ, McDermott B (2008) Recognizing and improving patient nonadherence to statin therapy. Curr Atheroscler Rep 10: 19–24. 18366981 10.1007/s11883-008-0004-4

[58] Bouchard M-H, Dragomir A, Blais L, Berard A, Pilon D, Perreault S (2007) Impact of adherence to statins on coronary artery disease in primary prevention. Br J Clin Pharmacol 63: 698–708. 10.1111/j.1365-2125.2006.02828.x 17214831 PMC2000596

[59] Wallach-Kildemoes H, Andersen M, Diderichsen F, Lange T (2013) Adherence to preventive statin therapy according to socioeconomic position. Eur J Clin Pharmacol 69: 1553–1563. 10.1007/s00228-013-1488-6 23588558

[60] Sugiyama T, Tsugawa Y, Tseng C-H, Kobayashi Y, Shapiro MF (2014) Different time trends of caloric and fat intake between statin users and nonusers among US adults: gluttony in the time of statins? JAMA Intern Med 174: 1038–1045. 10.1001/jamainternmed.2014.1927 24763487 PMC4307794

[61] Jørgensen T, Capewell S, Prescott E, Allender S, Sans S, Zdrojewski T, et al (2013) Population-level changes to promote cardiovascular health. Eur J Prev Cardiol 20: 409–421. 10.1177/2047487312441726 22514213

[62] Brunner EJ, Marmot MG, White IR, O’Brien JR, Etherington MD, Slavin BM, et al (1993) Gender and employment grade differences in blood cholesterol, apolipoproteins and haemostatic factors in the Whitehall II study. Atherosclerosis 102: 195–207. 10.1016/0021-9150(93)90162-N 8251006

[63] Gazmararian JA, Kripalani S, Miller MJ, Echt KV, Ren J, Rask K (2006) Factors associated with medication refill adherence in cardiovascular-related diseases: a focus on health literacy. J Gen Intern Med 21: 1215–1221. 10.1111/j.1525-1497.2006.00591.x 17105519 PMC1924753

[64] Hippisley-Cox J, Parker C, Coupland C, Vinogradova Y (2007) Inequalities in the primary care of patients with coronary heart disease and serious mental health problems: a cross-sectional study. Heart 93: 1256–1262. 10.1136/hrt.2006.110171 17344333 PMC2000947

