# Supplementary material for: Quantifying the Contribution of Statins to the Decline in Population Mean Cholesterol by Socioeconomic Group in England 1991 - 2012: A Modelling Study
Source: PLoS One. 2015 Apr 9;10(4):e0123112. doi: 10.1371/journal.pone.0123112 (PMC4391910; doi:10.1371/journal.pone.0123112)
Supplement: S1 Table — (DOCX) [file pone.0123112.s001.docx]

S1 Table. Statins effects and weights used for the estimation of the weighted mean E_w._

| **Chemical name** | **Strength** | **Total cholesterol reduction** | **Weights**  **(for the weighted mean** $\boldsymbol{E}_{\boldsymbol{w}}$**)** |
| --- | --- | --- | --- |
| Atorvastatin | 10 | 27.3% (24.9-30.2%) | 0.1046 |
| Atorvastatin | 20 | 32.7% (30.1-35.7%) | 0.0361 |
| Atorvastatin | 30 | 35.8% (34.8-36.7%)* | 0.0005 |
| Atorvastatin | 40 | 38.4% (34.6-42.3%) | 0.0350 |
| Atorvastatin | 60 | 41.0% (39.7-42.3%)* | 0.0005 |
| Atorvastatin | 80 | 42.8% (37.4-48.0%) | 0.0118 |
| Fluvastatin Sodium | 20 | 16.4% (14.6-18.4%) | 0.0006 |
| Fluvastatin Sodium | 40 | 20.7% (19.0-22.5%) | 0.0173 |
| Fluvastatin Sodium | 80 | 23.3% (20.6-25.9%) | 0.0163 |
| Pravastatin Sodium | 5 | 10.4% (0.7-20.2%) | 0.0001 |
| Pravastatin Sodium | 10 | 14.5% (12.5-16.2%) | 0.0038 |
| Pravastatin Sodium | 20 | 17.7% (16.9-18.9%) | 0.0111 |
| Pravastatin Sodium | 40 | 22.0% (20.7-23.0%) | 0.0106 |
| Rosuvastatin Calcium | 5 | 25.9% (24.7-27.6%) | 0.0114 |
| Rosuvastatin Calcium | 10 | 29.0% (27.8-30.6%) | 0.0214 |
| Rosuvastatin Calcium | 20 | 32.1% (30.9-33.6%) | 0.0042 |
| Rosuvastatin Calcium | 40 | 35.2% (34.0-36.6%) | 0.0012 |
| Simvastatin | 10 | 20.1% (18.9-21.7%) | 0.0477 |
| Simvastatin | 20 | 23.5% (22.4-25.0%) | 0.4261 |
| Simvastatin | 25 | 24.6% (24.3-25.0%)* | 0.0001 |
| Simvastatin | 40 | 27.0% (25.2-28.9%) | 0.2339 |
| Simvastatin | 80 | 30.4% (29.6-31.3%) | 0.0045 |
| Simvastatin & Ezetimibe | 20 | 23.5% (22.4-25.0%) | 0.0003 |
| Simvastatin & Ezetimibe | 40 | 27.0% (25.2-28.9%) | 0.0008 |
| Simvastatin & Ezetimibe | 80 | 30.4% (29.6-31.3%) | 0.0001 |
| * Values derived from linear regression with total cholesterol reduction as the dependent variable and the natural logarithm of strength as the independent one. The model was weighted against the inverse variance of the cholesterol reduction (not presented in this table) | | | |
