# Supplementary material for: Quantifying the Contribution of Statins to the Decline in Population Mean Cholesterol by Socioeconomic Group in England 1991 - 2012: A Modelling Study
Source: PLoS One. 2015 Apr 9;10(4):e0123112. doi: 10.1371/journal.pone.0123112 (PMC4391910; doi:10.1371/journal.pone.0123112)
Supplement: S2 Table — Overall, and by age group, sex and quintiles of index of multiple deprivation (QIMD) (1 = most affluent, 5 = most deprived) in England, 2011–12. Socioeconomic trends are also presented. Brackets contain 95% confidence intervals. (DOCX) [file pone.0123112.s002.docx]

S2 Table. Predicted mean total cholesterol (mmol/L) overall, and by age group, sex and quintiles of index of multiple deprivation (QIMD) (1 = most affluent, 5 = most deprived) in England, 2011-12. Socioeconomic trends are also presented. Brackets contain 95% confidence intervals.

|  | **18-34 (years)** | | **35-54** | | **55+** | | **Overall*** |
| --- | --- | --- | --- | --- | --- | --- | --- |
| **QIMD** | **Men** | **Women** | **Men** | **Women** | **Men** | **Women** |  |
| 1 (most affluent) | 4.81 (4.61 to 5.01) | 4.76 (4.60-4.92) | 5.60 (5.49 to 5.70) | 5.27 (5.16 to 5.39) | 5.64 (5.52 to 5.75) | 6.07 (5.97 to 6.17) | 5.50 (5.40 to 5.61) |
| 2 | 4.71 (4.56 to 4.86) | 4.46 (4.31 to 4.61) | 5.55 (5.40 to 5.69) | 5.24 (5.12 to 5.36) | 5.60 (5.48 to 5.72) | 6.04 (5.94 to 6.15) | 5.44 (5.33 to 5.54) |
| 3 | 4.64 (4.41 to 4.86) | 4.71 (4.53 to 4.88) | 5.73 (5.58 to 5.89) | 5.28 (5.17 to 5.40) | 5.47 (5.34 to 5.59) | 6.13 (6.00 to 6.25) | 5.43 (5.31 to 5.54) |
| 4 | 4.84 (4.65 to 5.02) | 4.61 (4.46 to 4.77) | 5.60 (5.42 to 5.78) | 5.42 (5.26 to 5.58) | 5.57 (5.41 to 5.74) | 5.99 (5.84 to 6.14) | 5.40 (5.28 to 5.51) |
| 5 (most deprived) | 4.79 (4.57 to 5.01) | 4.61 (4.46 to 4.76) | 5.58 (5.42 to 5.74) | 5.45 (5.30 to 5.60) | 5.38 (5.19 to 5.57) | 5.87 (5.69 to 6.05) | 5.28 (5.17 to 5.39) |
| **All** | 4.75 (4.66 to 4.84) | 4.62 (4.55 to 4.69) | 5.61 (5.54 to 5.68) | 5.32 (5.26 to 5.38) | 5.55 (5.49 to 5.61) | 6.03 (5.98 to 6.09) |  |
| **Slope of the trend** | 0.02 (-0.05 to 0.08) | -0.01 (-0.06 to 0.04) | 0.00 (-0.04 to 0.04) | 0.05 (0.01 to 0.09) | -0.05 (-0.10 to  -0.01) | -0.04 (-0.08 to  0.01) | -0.01 (-0.03 to  0.01)* |
| **P for trend** | 0.63 | 0.76 | 0.90 | **0.01** | **0.03** | 0.09 | 0.45* |
| * Adjusted for age and sex | | | | | | | |
