# Supplementary material for: Quantifying the Contribution of Statins to the Decline in Population Mean Cholesterol by Socioeconomic Group in England 1991 - 2012: A Modelling Study
Source: PLoS One. 2015 Apr 9;10(4):e0123112. doi: 10.1371/journal.pone.0123112 (PMC4391910; doi:10.1371/journal.pone.0123112)
Supplement: S1 Text — (DOCX) [file pone.0123112.s003.docx]

# Supporting the assumption of no statin effect in 1991-1992

In 1991-92 statins were not widely used, and only reserved for individuals with very high cholesterol. This is supported from the Health Survey for England 1991-92 data; utilisation of any lipid lowering medications, including statins, was 0.5% (95% CI: 0.3% to 1.0%) and the mean total cholesterol of the participants, on this medication, was 6.46 mmol/L (95% CI: 6.04 to 6.87). Moreover, statins available at that time were less effective and generally were prescribed in smaller strengths than today. So the E_w_ for 1991-92 would be much lower than the E_w_ for 2001-12. Finally statins are the most effective medication in lowering total cholesterol on individuals.

Let us consider the following scenario. All the lipid lowering medication users in 1991-92 were on statins and statins effectiveness was as high as we observed in 2011-12 (E_w_ for 1991-92 equal to E_w_ for 2011-12). Even in this extreme and highly unlikely scenario, the mean total cholesterol of the population for 1991-92 with the effect of statin removed, would be 5.87 mmol/L (95% CI: 5.83 to 5.91). Not much different from the observed one of 5.86 mmol/L (95% CI: 5.82 to 5.90). Therefore, we consider the bias from our decision to ignore any possible statin effect in 1991-92 negligible.
